# Smart Bioimpedance Spectroscopy Device for Body Composition Estimation

**DOI:** 10.3390/s20010070

**Published:** 2019-12-21

**Authors:** David Naranjo-Hernández, Javier Reina-Tosina, Laura M. Roa, Gerardo Barbarov-Rostán, Nuria Aresté-Fosalba, Alfonso Lara-Ruiz, Pilar Cejudo-Ramos, Francisco Ortega-Ruiz

**Affiliations:** 1 Biomedical Engineering Group, University of Seville, 41092 Seville, Spain; jreina@us.es (J.R.-T.); lroa@us.es (L.M.R.);; 2 Nephrology Service of the Virgen Macarena University Hospital in Seville, 41009 Seville, Spain; narestef@senefro.org (N.A.-F.); alararnet@hotmail.com (A.L.-R.); 3 Medical-Surgical Unit of Respiratory Diseases, University Hospital Virgen del Rocío, 41013 Seville, Spain; mariap.cejudo.sspa@juntadeandalucia.es (P.C.-R.); francisco.ortega.sspa@juntadeandalucia.es (F.O.-R.); 4 Biomedical Research Center in Network (CIBER) of Respiratory Diseases (CIBERES), 28029 Madrid, Spain

**Keywords:** bioimpedance spectroscopy, body composition estimation, cole model, dispersion, parameter identification

## Abstract

The purpose of this work is to describe a first approach to a smart bioimpedance spectroscopy device for its application to the estimation of body composition. The proposed device is capable of carrying out bioimpedance measurements in multiple configurable frequencies, processing the data to obtain the modulus and the bioimpedance phase in each of the frequencies, and transmitting the processed information wirelessly. Another novelty of this work is a new algorithm for the identification of Cole model parameters, which is the basis of body composition estimation through bioimpedance spectroscopy analysis. Against other proposals, the main advantages of the proposed method are its robustness against parasitic effects by employing an extended version of Cole model with phase delay and three dispersions, its simplicity and low computational load. The results obtained in a validation study with respiratory patients show the accuracy and feasibility of the proposed technology for bioimpedance measurements. The precision and validity of the algorithm was also proven in a validation study with peritoneal dialysis patients. The proposed method was the most accurate compared with other existing algorithms. Moreover, in those cases affected by parasitic effects the proposed algorithm provided better approximations to the bioimpedance values than a reference device.

## 1. Introduction

Bioimpedance methods are common techniques for body composition (BC) estimation. They do not have the restrictions of solution methods [1,2], are simple, safe and noninvasive, and provide more accurate estimations than anthropometric methods [1,3]. In addition, bioimpedance measurements have numerous practical advantages that have favored their rapid development [1]: the instrumentation is portable, it is a non-invasive technique, safe and easy to perform, results are obtained immediately, and measurements can be repeated as often as desired, with great inter-observer reproducibility. Thanks to bioimpedance techniques, it is possible to obtain an estimation of body fluids and nutritional status in both normal and disease states. The clinical utility of BC analysis through bioimpedance techniques has been demonstrated in numerous studies: in nutrition [4,5], during pregnancy and lactation [6], in the evaluation of the risk of various pathologies [7,8], as a marker or direct cause of disease [9,10], during the process of decision making in an illness [11], aging [12] or rehabilitation processes [13], as a complement to the diagnosis and follow-up of conditions related to the cardiovascular system [14,15], in oncology [16] and even in sports science [17], among others.

Despite the advances in the clinical application of bioimpedance, there are still some challenges to be solved, such as the integration of devices into e-Health systems supporting remote user monitoring [18]. In addition, the complexity of bioimpedance systems is usually quite high (current injection, voltage measurement, demodulation, processing, etc.) and the use of high-frequency signals (tens to hundreds of kHz) generally requires high power consumption, so new challenges arise for the optimization of hardware in size, energy efficiency, robustness and precision [1,19]. On the other hand, although the bioimpedance spectroscopy technique has proven to be more precise and robust than the single frequency technique, most devices base their operation on the measurement on a single frequency [1,20].

Other issues are related to the models and algorithms used to estimate the BC from bioimpedance measures. A key point for an adequate BC estimation by bioimpedance analysis is a correct parameter identification of the bioimpedance model, usually the Cole model [1,21,22,23,24,25,26,27,28,29,30,31]. Sometimes, this parameterization is performed without directly measuring the impedance values [21,22,23]. Other authors use only the impedance modulus when performing parameter identification [24,25,26]. However, the techniques that achieve a smaller error use both the real and the imaginary parts of the impedance [25]. Bioimpedance model identification is normally obtained by means of nonlinear Least Squares (NLLS) methods, which aim at obtaining the best coefficients for the Cole model that fits the curve minimizing the squared sum of the error between the measured data and the modeled values [23,25,27,28]. Other methods employ a stochastic resolution approach like the particle-swarm optimization algorithm [29,30], bacterial foraging optimization method [30] or genetic algorithms [30,31]. These methods do not often solve analytically the problem of parameter identification, but use complex algorithms of successive approximations that can only be executed off-line on personal computers.

However, the biggest problem in the parameter identification process is the occurrence of some kind of perturbation, noise or parasitic effect. These effects can significantly affect the parameters obtained, inducing errors in the estimation of BC [25,27]. The vast majority of the algorithms proposed in the literature are applied to the single-dispersion Cole model, although it has been proven that the extended Cole model is more robust against possible parasitic effects [2,32].

In this context, the purpose of this paper is to describe a first approach to a smart bioimpedance spectroscopy device for body composition estimation. A first part of the study is focused on the hardware and the research, development and validation of the measurement instrumentation, processing and wireless sending of bioimpedance values. A second part is centered on the software. In this context, this paper proposes a simple but robust method for the extended Cole model parameter identification. This model is further enriched with a scheme that incorporates the effect of three dispersions. The two additional dispersions and the phase delay allow to include in the modeling external alterations with time constants different from those of the body environment, both in the low and high frequency ranges, as well as temporary delays caused by the cables and the hardware of the devices.

## 2. Materials and Methods

The development of the bioimpedance device has followed a user-centered, based on standards, and modular design methodology to facilitate the integration of new technologies and functionalities. Cadence OrCAD software package (version 16.0) was used for the electronic design. For the implementation of printed circuit boards, ProtoMat S62 milling machine from LPKF was used. The processing code for the estimation of bioimpedance values was developed in assembly language for its embedded programming in the processing subsystem of the smart device.

### 2.1. Monitoring System Architecture

The monitoring is performed in a distributed way through the following devices arranged in a master-slave architecture:

- **Smart bioimpedance device**: It is a portable device capable of carrying out bioimpedance measurements in multiple configurable frequencies, processing the data to obtain the modulus and the bioimpedance phase in each of the frequencies, and transmitting the processed information wirelessly.
- **Personal monitoring device** (master): Data acquired by the smart bioimpedance device are wirelessly transmitted to a smartphone that performs a second processing to estimate the BC from the identification of parameters of a three-dispersion bioimpedance model. Besides, by adapting the model described in [3] the personal monitoring device provides an estimation of the following parameters: extracellular water (ECW), intracellular water (ICW), total body water (TBW), fat mass (FM) and fat free mass (FFM). The processing algorithms are implemented in Java by a mobile application for Android operating system. This device also serves as user interface and communications gateway for data storage and remote management of the information.

In the context of the present work, smart sensor is understood as the one that not only has sensing capabilities for the measurement of certain variables, bioimpedance in this case, but is also equipped with a communications unit, is capable of carrying out of a pre-processing of the information acquired, and has customization capabilities. In this case, pre-processing deals with the estimation of the modulus and phase of the bioimpedance at configured frequencies, and personalization is related to the ability to adapt the operation of the device to different frequencies and operation modes. This allows the device to be used in other applications where the measurement of bioimpedance at multiple frequencies is appropriate. In addition, the operation of the device is supported by a preliminary calibration procedure, which will be explained in Section 2.4, which further accentuates the intelligent character of the device. Thus, the processing load is distributed between the smart bioimpedance device and the personal monitoring device, what provides numerous technical advantages, such as a reduction in global energy consumption.

### 2.2. Architecture of the Smart Bioimpedance Device

**Figure 1:**
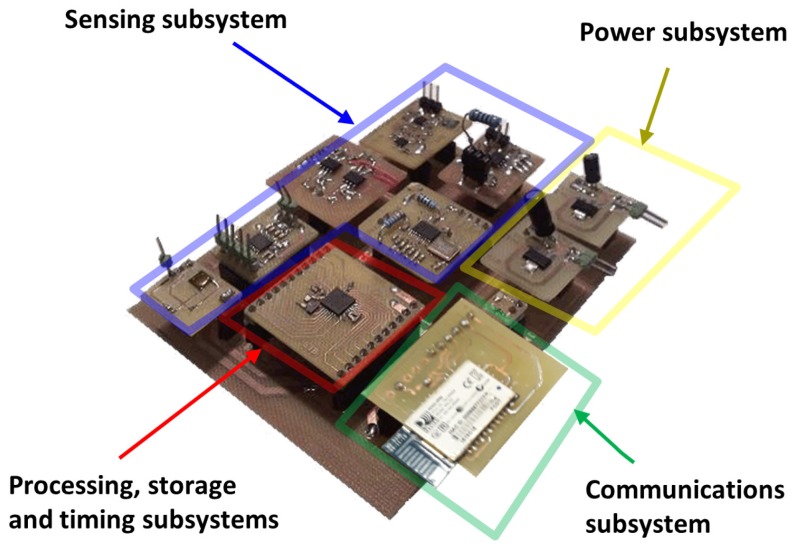
Prototype of the bioimpedance device.

**Figure 2:**
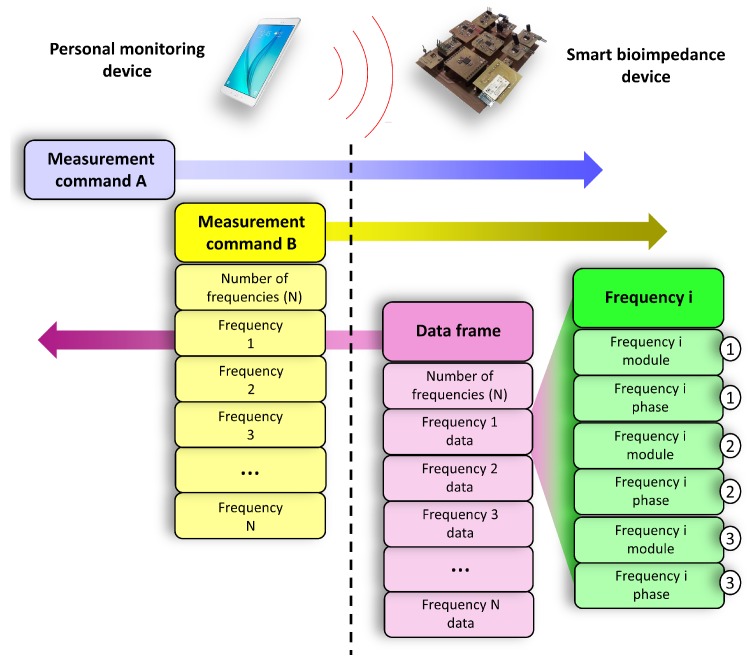
Data flow between the smart bioimpedance device and the personal monitoring device.

The bioimpedance device architecture follows a modular design scheme described in the form of subsystems (see Figure 1):

- **Sensing subsystem**: Which encompasses the necessary hardware to perform bioimpedance measurements. The referred subsystem generates an alternating electric current of known amplitude to be injected into the human body through two electrodes (distal electrodes). By means of two other electrodes located in the current path (proximal electrodes) the sensing subsystem makes a measurement of the voltage generated by the circulation of the current. Two 150-cm cables connect the smart bioimpedance device with the electrodes: a cable for the electrodes to be placed on the hand and another one for the electrodes to be placed on the foot. Each cable has two lines, and each line is shielded to reduce the effects of electromagnetic noise. The cables end in crocodile clips for the connection with the electrodes.
- **Processing subsystem**: Integrates the hardware, software and firmware elements of the smart bioimpedance device that are applied in the processing for the estimations of the module and phase of bioimpedance in each of the frequencies. Frequencies can be configured remotely by sending a command. The processing subsystem is also responsible for the correct activation and configuration of the different modules of the sensing subsystem each time a new bioimpedance measurement is performed. Thus, the energy consumption of the smart bioimpedance device is reduced, deriving the different modules from the sensing subsystem to low consumption modes of operation when they are not necessary. The processing subsystem is implemented in a microprocessor with an 8-bit arithmetic-logic unit that operates at 4 MHz (PIC18LF2431 microprocessor from Microchip). Pre-processing algorithms are programmed in the microprocessor’s flash memory, which has a capacity of 16 Mbytes.
- **Communications subsystem**: RN42 module from Microchip has been used for the development of the wireless communications of the smart bioimpedance device according to the SPP profile of Bluetooth v2.1 standard. Communications are bidirectional to allow the sending of results of the processing subsystem in one way, and the remote configuration of the smart sensor by sending commands in the other way. According to the communications protocol for the device control, the personal monitoring device starts a bioimpedance measurement by sending a command (Measurement command A in Figure 2). The frequencies are pre-configured in the smart bioimpedance device, although it is also possible to define the number and value of the frequencies to be acquired by sending a special command (Measurement command B). Once the multifrequency measurement is finished, the smart bioimpedance device sends a response data frame containing the frequencies (Data frame), the modulus and phase of the bioimpedance following the data structure described in Figure 2. It should be mentioned that for each frequency, three consecutive estimations of the bioimpedance modulus and phase are performed, which are also sent in the data frame to provide a more robust measurement. Depending on the dispersion of bioimpedance values, the personal monitoring device may decide to use the average value of the three measurements or discard the frequency in the identification of parameters of a three-dispersion bioimpedance model.
- **Data storage subsystem**: It is responsible for the correct storage of the data used by the smart bioimpedance device. To develop the data storage subsystem, the 768-byte SRAM memory and the 256-byte EEPROM memory of the PIC18LF2431 microprocessor are used.
- **Timing subsystem**: Which deals with the maintenance of a real-time timing system and the assignment to each measurement of the time in which they were made for registration and subsequent monitoring. Such subsystem is also responsible for notifying the processing subsystem of the instants for carrying out operations whose timing has been pre-configured. An external crystal of 32.768 kHz and one of the microprocessor timers are used to manage its operation. The timing subsystem is also implemented in the program code of PIC18LF2431 microprocessor by managing the interrupts of one of the microprocessor’s internal timers. An external crystal clock of 32.768 KHz is used to precisely define the timing with a real-time clock.
- **Energy subsystem**: Provides the necessary supply voltages for the proper operation of all subsystems. A lithium polymer battery with 3.7 nominal voltage provides power for device operation. BQ21040 charger device from Texas Instruments is used to manage the battery charge. A LD1117S33TR regulator from STMicroelectronics provides 3.3 V supply voltage for the digital electronic components (microprocessor, transceiver, etc.). The TPS65133 DC-DC converter from Texas Instruments is employed to supply the ±5.0 V required for the sensing subsystem. The microprocessor can turn off the analog electronics of the sensing subsystem through a line that controls the operation of TPS65133 component, in order to reduce energy consumption.

### 2.3. Architecture of the Sensing Subsystem

The sensing subsystem is the result of a novel transformation of the generic detection scheme of demodulation of signals in quadrature [1,33]. As an advantage over other systems, the scheme proposed uses a single multiplier, which allows reducing costs, also avoiding errors derived from possible differences between components.

**Figure 3:**
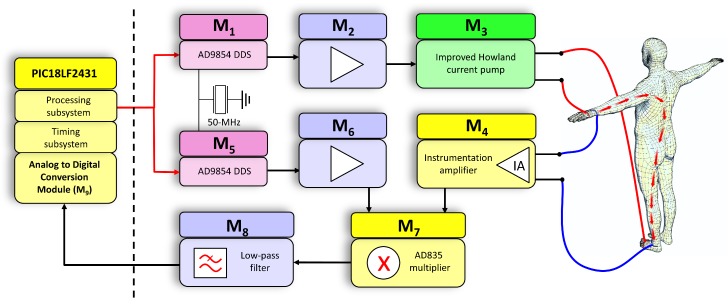
Architecture of the sensing subsystem.

The sensing subsystem is broken down into the following functional modules, as shown in Figure 3:

- **Injection signal generation module ($M_{1}$)**: This is a programmable oscillator that uses the Direct Digital Frequency Synthesis (DDS) technique. This module generates a sine voltage waveform ($S_{1}$) with fixed amplitude ($A_{1}$). The frequency ($f_{1}$) of the signal ($S_{1}$) can be configured to scan bioimpedance measurements at different configurable frequencies. AD9854 high-speed DDS from Analog Devices has been used to generate a high-precision sine waveform capable of being configured to generate any frequency from 0.19 Hz to 5 MHz, with a resolution of 0.19 Hz and a stability of 40 ppm. A 50-MHz clock was used to provide the necessary synchronization for the operation of the DDS. The PIC18LF2431 microprocessor controls the frequency and phase of the signal generated by the DDS through a serial data interface. This scheme gives the system great flexibility to generate signals of different frequency. As mentioned previously, the frequencies of this sweep can be configured remotely by means of a command so that they usually take any value between 1 kHz and 1 MHz in a body bioimpedance measurement application (lower frequencies are not recommended to avoid interfering with the biological signals and higher frequencies are more sensitive to electromagnetic noise [1]) although depending on the application this range can be extended to both low and high frequencies (from 0.19 Hz to 5 MHz). As also mentioned, the number of frequencies of the bioimpedance measurement scan is also a configurable parameter, although a single-frequency analysis can be performed as well. An input line to the DDS, also controlled by the microprocessor, allows it to be configured in a low-energy mode, in which the operation of the device is suspended and the signal generation process is canceled. Signal discontinuities due to digital sampling are smoothed through an low-pass filter inside the DDS with a cutoff frequency high enough not to affect the generated signals.
- **Injection signal amplification module ($M_{2}$)**: Amplifier with ($A_{2}$) gain applied on signal ($S_{1}$) to generate signal ($S_{2}$). Since the output signal of the DDS is very weak (about 0.6 Vpp), an amplifier based on operational amplifiers (OpAmps) is used to increase the signal amplitude. This module also has the function of decoupling the DDS from the voltage-current conversion module (the DDS has an output resistance of 50 Ω), providing a suitable voltage level for its operation.
- **Voltage-current conversion module ($M_{3}$)**: Transconductance amplifier that converts the voltage signal at the output of the injection signal amplification module ($S_{2}$) into a current signal ($S_{3}$), injected into the human body through the distal electrodes. The amplitude ($A_{I}$) of the injected current intensity has a constant value, preset to comply with international safety standards [1]. In addition, the referred current amplitude is independent of the impedance of the human body, the impedance of the electrodes and the frequency at which the measurement is made. An improved Howland current pump [34,35] has been used for its implementation, due to its precision and stability characteristics, through OpAmps. The current source was configured to generate an effective current of 0.4 mA, well below the international safety limits for this type of applications [36].
- **Signal detection module ($M_{4}$)**: Instrumentation amplifier that applies a gain ($A_{4}$) to the voltage detected through the proximal electrodes ($S_{B}$), generating the signal ($S_{4}$). The input impedance of the instrumentation amplifier is very high so that the voltage drop in the proximal electrodes can be considered negligible. The instrumentation amplifier has been implemented through discrete low-noise OpAmps, providing a gain of 2, a bandwidth of 45 MHz, a common-mode rejection ratio (CMRR) of 94 dB and an input impedance of 1 MΩ.
- **Internal signal generation module ($M_{5}$)**: It generates an internal sine voltage waveform ($S_{5}$) with the same amplitude value ($A_{1}$) and frequency ($f_{1}$) as the signal ($S_{1}$), but with a phase difference ($\Phi_{5}$) that alternates its values between 0${}^{\circ }$ and 90${}^{\circ }$ to measure the in-phase and quadrature components. Another DDS with the same characteristics as ($M_{1}$) is used for its implementation. This is a relevant aspect of the present design, which allows precise control of the phase measurement and, together with the possibility of controlling the measurement frequency, allows the device to be used for different applications. This phase and frequency control is the basis of the calibration procedure described in Section 2.4. In addition, the proposed scheme allows the use of only a single multiplier to perform the demodulation of signals in phase and quadrature, which is the principle used for the measurement of the biompedance modulus and phase [1,37]. The measurement process is carried out in two stages: first, a frequency sweep in which the parameter $\Phi_{5}$ takes the value 0${}^{\circ }$ is carried out for the evaluation of in-phase components; second, a subsequent frequency sweep with $\Phi_{5}$ set to 90${}^{\circ }$ is performed to measure the quadrature-phase components. The processing subsystem is responsible for configuring this transition in the phase, waiting for the required time for the signals to stabilize before the measurement is performed. The use of two DDS caused a continuous drift of the phase lag between the signals generated by both DDS over time (the phase lag did not remain fixed, but increased or decreased over time), making difficult the phase control between the signals generated by both DDS. This effect was more evident the higher the frequency. The cause of the drift in the phase came from the difference in the frequency of the crystal clocks that controlled each DDS, however small this difference was. This problem was solved by employing a single 50-MHz crystal used as time reference for both DDS. Thus, both devices have exactly the same frequency and the offset between the signals of both modules remains constant over time.
- **Internal signal amplification module ($M_{6}$)**: Amplifier with ($A_{6}$) gain applied on signal ($S_{5}$) to generate signal ($S_{6}$). The function of this module is to decouple the module ($M_{5}$) from the module ($M_{7}$), also adapting the voltage of the sine waveform to adequate levels of operation. It has been implemented using discrete OpAmps.
- **Multiplier module ($M_{7}$)**: This module generates signal ($S_{7}$) as a result of the multiplication of signal ($S_{4}$) and signal ($S_{6}$). A AD835 full complete four-quadrant multiplier from Analog Devices has been used for this purpose. The resulting signal ($S_{7}$) is formed by the superposition of a sinusoidal waveform with frequently ($2\cdot f_{1}$) and a continuous level that depends on the phase lag between the input signals and their amplitudes. Thus, the continuous level of in-phase and quadrature components allow to calculate the bioimpedance modulus and phase by means of a coherent demodulation process [1,37].
- **Filtering module ($M_{8}$)**: It is an active second-order low pass filter based on OpAmps with a cut-off frequency of 13.8 Hz that extracts the continuous component ($S_{8}$) from signal ($S_{7}$). The filter cutoff frequency is low enough to keep the signal ripple below 1% with respect to the continuous level at all operating frequencies.
- **Analog to Digital Conversion Module ($M_{9}$)**: This module is responsible for converting the analog signal ($S_{8}$) into digital signals with which the processing subsystem can operate. It is responsible for calculating the bioimpedance modulus and phase following the standard procedure of demodulation of signals in quadrature [1], taking into account the characteristics of the proposed scheme. For its implementation, one of the 10-bit analog-digital converters of the microprocessor has been used, setting the maximum voltage to provide a resolution of 1.17 mV.

### 2.4. Addressing Parasitic Effects

A parasitic effect is an undesired behaviour in a system component that moves it from ideal performance. Although a bioimpedance measurement device for human body is more robust against noise and parasitic effects compared with other electrophysiological systems, such as ECG, it is not free of them [38]. These effects are primarily related with undesired capacitances, but also with resistive and inductive phenomena.

In the context of bioimpedance technology, electrodes are essential components, as they provide the required electro-chemical interface with the human body for the injection of electric current and voltage measurement. However, they also become an important error source due to the polarization of the electrode-skin contact, capacitive phenomena, issues derived from a high impedance in this contact or variations in their values due to pressure fluctuations or movement-related artefacts [39,40,41].

To reduce the possible parasitic effects associated with electrodes, in this work we used pre-gelled adhesive Ag/AgCl non-polarizable electrodes, to reduce the contact electrode-skin impedance and avoid polarization-related effects. A rectangular shape with 1.5 cm × 6 cm was selected to maximize electrode surface, thereby reducing their impedance. These electrodes were placed transversally to the electric current pathway in the wrist and ankle of the same side of the body. For the hand, the distal electrode was placed on the back, at the level of metacarpus, and the proximal electrode extending from the carpus. For the foot, the distal and proximal electrodes were placed on the back, metatarsus and tarsus, respectively. These electrodes are adapted to body shape and a side strap eases attachment to the bioimpedance cables through crocodile clips.

To reduce the effects of electrode drifts, the subjects under test remained 10 min in horizontal position with the pre-gelled electrodes attached, in order to allow the stabilization of sweat glands and gel temperature [42]. This procedure also favored the uniform distribution of body fluids throughout the entire body, preventing accumulation in the lower limbs. On the other hand, to protect the subject in case of failure, a high-pass filter based on a dc-blocking capacitor was included in the electric pathway. As referred in Section 2.2, the device has been conceived to be battery-powered.

As aforementioned, it is worth emphasizing that a four-electrode setup was considered, which injects current through the distal electrodes and voltage is measured through the proximal electrodes using a high input impedance instrumentation amplifier [1]. In this way, current circulating in the measurement electrodes is negligible and the electrode impedance can be neglected compared to body impedance [43].

Regarding the measurement instrumentation, a primary error source is the current generator. To reduce possible adverse effects, a current source based on an improved Howland current pump [34,35] was used, which is stable and accurate in the full range of the considered frequencies and independent of the particular human body bioimpedance values [33]. This current source was designed to exhibit a very high output impedance, low stray capacitance [42] and a bandwidth large enough not to affect signals up to 1 MHz. On the other hand, to reduce possible measurement artefacts related to the operation frequency harmonics [44], a precision DDS was used, which generates high-quality sinusoidal waves.

Another relevant component is the instrumentation amplifier, which exhibits a high input impedance, a CMRR above 80 dB, and a bandwidth large enough to provide accuracy and robustness against noise in the full measurement band [44,45,46].

To reduce the issues related to electromagnetic noise and interferences, both the cables and sensing subsystem were shielded. Besides, accuracy and noise of the electronic components were evaluated individually for the different modules of the sensing subsystem. These modules were designed and tested individually, both stage by stage and globally, following a design, development and validation cycle, until the robustness and stability requirements were met for different loads and frequencies.

Error sources related to hardware can be removed by considering an appropriate calibration procedure that includes the nonlinearities of the electronic components and their deviations in magnitude and phase, together with the effects of cables and electrodes [47,48,49]. This calibration has been performed using the following semi-automated procedure:

- For the calibration of the device a series resistor pattern set was used, which allowed testing values in the range from 100 Ω up to 1050 Ω with increments of 50 Ω.
- The device was configured to evaluate sequentially 16 phase values, modeling the phase shift between the output signal and the internal reference signal. This way, phase could be swept in increments of 5${}^{\circ }$ from 0${}^{\circ }$ to 75${}^{\circ }$.
- For each resistor value of the calibration pattern and each evaluated phase, an automatic full frequency sweep was performed, and changing the resistance value after all phases were evaluated.
- The full process was repeated 5 times to provide a better robustness against measurement noise.
- The measured values were compared against the reference values to provide a fitting of the calibration curves in terms of frequency, impedance modulus and phase.

**Figure 4:**
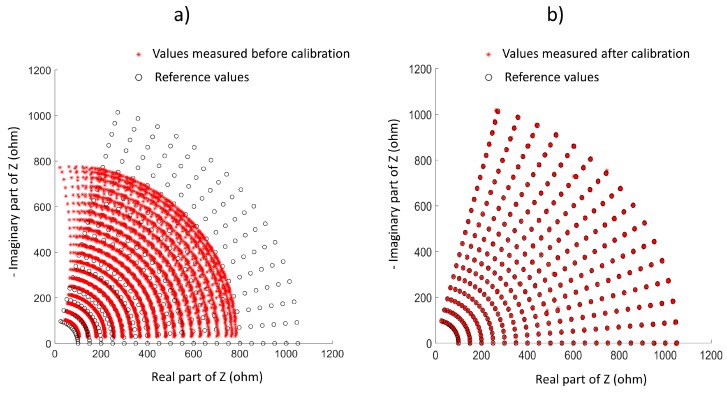
Example of measurements used in calibration: (**a**) before calibration, (**b**) after calibration.

For the sake of illustration, Figure 4 shows the bioimpedance values obtained before and after calibration. We note that the calibration pattern was purely resistive, and phase tuning was performed by modifying phase using the hardware. Against the common calibration procedure for devices capable of measuring phase, a resistive network was used for calibration instead of mixed resistors and capacitors [50,51,52]. The proposed method allows a better control of phase, avoiding possible nonlinearities in the capacitive components of the calibration pattern. Finally, and only for validation purposes, a common impedance network composed of resistors and capacitances was used, to be described in Section 2.7, and setting a target error below 1%. As temperature and aging conditions affect all electronic devices, such as OpAmps and resistors, the proposed method represents an approach to calibration that can be of interest for clinical use [42].

As previously said, the correct identification of single dispersion Cole model is essential for bioimpedance spectroscopy technique. This dispersion is related to β dispersion that occurs in biological media [53], usually in the 10–100 MHz range as a consequence of the polarization effects in cell membranes [54]. In body measurements, α dispersion, in the usual range of 10 Hz to 10 kHz, and γ dispersion, in the gigahertz range, use to be negligible, such that they are not taken into consideration in bioimpedance models [55,56]. The error in the estimation of the model is reduced if the number of measurement frequencies is increased [57], because the model that provides a best fit to all frequencies provides a safeguard against noise at any frequency. For this reason, a high number of frequencies has been used in the two studies carried out: 20 points used in the hardware validation study described in Section 3.1 and 50 for the software validation study described in Section 3.2 (all frequencies are in kHz): 5, 6, 7, 8, 9, 10, 11, 12, 13, 14, 15, 16, 18, 20, 23, 25, 28, 31, 35, 39, 43, 50, 54, 60, 67, 75, 83, 93, 100, 115, 128, 143, 159, 177, 200, 220, 245, 273, 304, 339, 378, 421, 469, 500, 582, 649, 723, 806, 898, 1000. The points that were only used in the hardware validation study are underlined. Although the device can measure at any frequency between 0.19 Hz and 5 MHz, these points were selected to allow the comparison with the reference device in the evaluation studies.

As the different frequencies cannot be evaluated at the same time and a frequency sweep is required, a possible noise source is the movement artefacts during the multiple-frequency measurement process. In order to avoid this problem, the volunteers were advised to stay still during the measurement time. The required time to complete the full range of frequency measurements was reduced to the mimimum allowing error-free impedance modulus and phase, which was obtained in 10 s. Frequencies reporting bioimpedance values far away from the model were discarded, considering that they were consequence of some artefact, resuming the model identification procedure with the remaining impedance set.

An imperfect electrode-skin contact (e.g., partially separated or improperly conditioned skin) or a defective gel can be associated with an increase of contact impedance [58]. When the electrode–skin contact impedance takes a high value for any reason, comparable to the input impedance of the instrumentation amplifier, a low–frequency dispersive effect may arise. In general, dispersive effects occur when charge storage is superimposed to a dissipation mechanism [59]. This phenomenon has been evidenced when dry electrodes are used, which can present impedances several magnitude orders higher than pre-gelled Ag/AgCl electrodes [60], and the capacitive effect of the electrode-skin contact takes a higher relevance. Electrode polarization is a parasitic effect consequence of charge accumulation in the electrode/electrolyte interface. This charge can increase impedance corrupting measurements at low frequencies [61]. This effect has been reported with the modeled parasitic electrode impedance evaluated in [62], showing significant alterations at low frequencies.

For frequencies above 100 kHz, the major error source in bioimpedance measurements is the parasitic impedance associated with the return path of the human body with the ground plane [63]. This capacitance can be in the range of tens of pF to several nF, and translates into a time constant related to the capacitance value and the sum of the overall system resistances (both measured and parasitic) [63,64]. A hook effect can be observed in the Cole diagram representation at high frequencies [65], which superimposes to the linear phase shift with frequency derived from cable lengths and hardware. This effect can be regarded as a dispersive artefact that can be relevant above 100 kHz. Many authors discriminate this parasitic effect by including a parasitic shunt capacitance in the bioimpedance model [66], however, we address this effect by considering a third dispersion at high frequency, above the main dispersion.

To solve these issues at low and high frequencies a procedure is proposed in Section 2.6 from the three-dispersion model described in Section 2.5.

### 2.5. Proposed Artefact-Aware Bioimpedance Model

As mentioned in Section 1, a widespread bioimpedance model is the single-dispersion Cole model, which can be expressed by the following Equation [2]:

(1) $$Z=R_{\infty }+\frac{R_{0}-R_{\infty }}{1+{\left[j\omega \tau \right]}^{1-\alpha }}\;,$$

where $R_{0}$ is equivalent to the extracellular resistance ($R_{e}$), $R_{\infty }$ is the shunt association of $R_{e}$ and the intracellular resistance ($R_{i}$); $\tau =\left(R_{e}+R_{i}\right)C_{m}$ is the time constant related to the characteristics of the cell membrane and the values of the resistors ($C_{m}$ is the membrane capacitance), ω=2πf, *f* is the frequency and α is a parameter (0≤α≤1) that defines the frequency behavior of the dispersion.

An extension of the model that includes a first dispersion at low frequency and a third dispersion at high frequency is proposed, not to account for the α and γ dispersions of bioimpedance, but to model possible parasitic effects beyond the β dispersion of bioimpedance (main or second dispersion in the proposed model). As mentioned in Section 2.4, low frequency dispersion is intended to model possible artefacts related to the electrode-skin contact, while high frequency dispersion tries to incorporate the effects derived from the parasitic capacitance that the human body establishes with the external ground plane. To the knowledge of the authors, a three-dispersion model that tries to take into account possible parasitic effects has not been explicitly considered in bioimpedance measurement systems:

(2) $$Z=R_{\infty }+\frac{\Delta R_{1}}{1+{\left[j\omega \tau_{1}\right]}^{1-\alpha_{1}}}+\frac{\Delta R_{2}}{1+{\left[j\omega \tau_{2}\right]}^{1-\alpha_{2}}}+\frac{\Delta R_{3}}{1+{\left[j\omega \tau_{3}\right]}^{1-\alpha_{3}}}\;,$$

in which:

(3a) $$\begin{array}{c} R_{\infty }=R_{\infty ,1}+R_{\infty ,2}+R_{\infty ,3} \end{array}$$

(3b) $$\begin{array}{c} \Delta R_{k}=R_{0,k}-R_{\infty ,k}\;,k=1,2,3 \end{array}$$

The proposed model also includes the effects of the delays in the signals caused by the electrodes, the wires and the hardware, which can be modeled by a phase delay ($T_{d}$) that linearly increases with frequency (extended Cole model):

(4) $$Z^{\prime }=Ze^{-j\omega T_{d}}$$

It should be noted that clinical bioimpedance measurement devices usually correct the typical phase delay of the device using a factory calibration so that the modeled phase delay shows the variations from the typical phase delay.

### 2.6. Description of the Proposed Algorithm

This paper proposes a method that solves the parameter identification of the extended Cole model with three dispersions in a quasi-analytic manner. It will be referred to as the Extended Cole model with 3 Dispersions (EC3D) algorithm. The procedure is based on an algorithm that minimizes the error between the measured bioimpedance values and those that could be found with the model at the corresponding frequencies. The main novelties of the algorithm are the following:

- It is an algorithm able to identify the parameters of a Cole model of three dispersions. To the authors’ knowledge, no procedure or algorithm has been published that explicitly solves the parameter identification in a three-dispersion bioimpedance model. In addition, and as detailed in Section 2.5, the first and third dispersion of the proposed model are not related to bioimpedance values, but to artefacts that may appear at low and high frequencies, and if they are not removed or taken into account, can greatly affect in the parameters identification process.
- It is a low-computational-load algorithm capable of being efficiently solved in a low-performance microprocessor thanks to a novel approach that analytically solves the model on an iterative scan in a single parameter ($T_{d}$).

**Figure 5:**
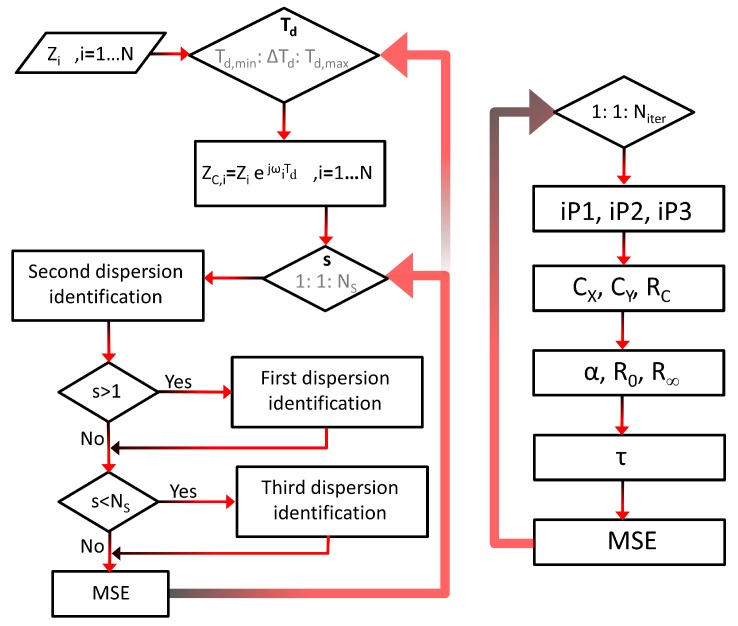
Flow chart of the proposed algorithm.

**Figure 6:**
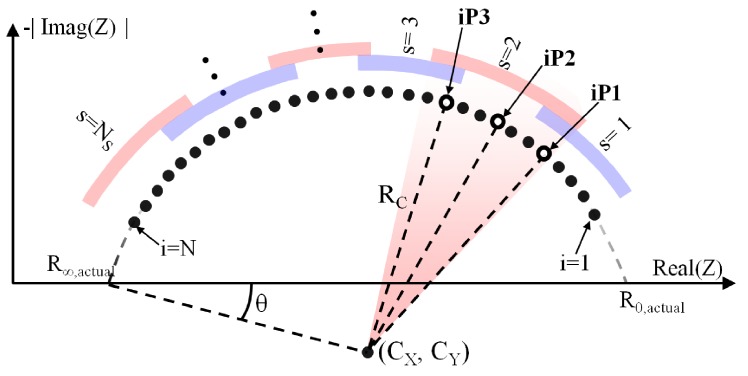
Singular points and geometric variables of the proposed algorithm.

The algorithm steps are detailed next (see Figure 5 and Figure 6):

1. Load *N* complex measured impedance values (real and imaginary parts), in *N* consecutive frequencies.
2. The base of the processing is an iterative search of the solution for a single parameter of the model, the parameter $T_{d}$ (from $T_{d,min}$ to $T_{d,max}$ in increments of $▵T_{d}$). The main advantage of this approach is that the search of the solution depends only on one parameter, such that the required number of iterations is appreciably reduced.
3. For each value of $T_{d}$, the bioimpedance values are corrected according to the expression of $Z_{C,i}$, where index *i* refers to the frequency. $Z_{C,i}$ is the impedance that would be obtained if the influence of the delay modeled by $T_{d}$ were removed.
4. The impedance values are grouped into $N_{S}$ sectors, which might overlap. The algorithm will cover the different bioimpedance sectors to find the best fit with the second dispersion, where *s* indicates the number of the sector.
5. Identification of the Cole model (second dispersion) that best fits the impedances of sector *s*.
6. If *s* is higher than 1, identification of the Cole model (first dispersion) that best fits the result of subtracting the second dispersion to the corrected impedances $Z_{C,i}$, considering only the lower frequencies.
7. If *s* is lower than $N_{S}$, identification of the Cole model (third dispersion) that best fits the result of subtracting the second dispersion to the corrected impedances $Z_{C,i}$, considering only the higher frequencies.
8. Calculation of the mean square error (MSE) between the measured impedance values and those obtained from the three-dispersion model. If this error is the smaller until then, the parameters evaluated are proposed as the actual solution of the model. Finally, the algorithm loop is closed with the execution of step 2.

The identification of the Cole model (single dispersion) can be summarized in the following procedure, which is repeated $N_{iter}$ times. A higher $N_{iter}$ increases the probability of finding adequate parameters, but also increases the execution time.

9. The impedance values analyzed are grouped in sets of three elements in each iteration: one corresponding to a low frequency (index iP1), another to a medium frequency (index iP2) and the last one to a high frequency (index iP3). Index values are pre-configured to sweep all frequencies in a pseudo-random way, avoiding the repetition of triplets.
10. If the real and the imaginary parts of the absolute value of the bioimpedance are plotted for each of the sets, three points are obtained in the first quadrant. When three points are defined in a plane, it is possible to define a fourth point which is equidistantly located with respect to the other three. This property is used to calculate the radius $R_{C}$ and the point $C_{X},C_{Y}$ corresponding to the center of the circle that matches the three points, taking into account that the center corresponds to the fourth point and the radius with the distance mentioned.
11. The values of the parameters α, $R_{0}$ and $R_{\inf}$ for each of the sets of three impedances are calculated from the angle θ between the real axis, the cut-off point of the circumference closest to the origin, and the center of the semicircle.
  (5a) $$\begin{array}{c} \alpha =\frac{2\theta }{\pi } \end{array}$$
  (5b) $$\begin{array}{c} R_{0}=X_{C}+R_{C}\cos\theta \;, \end{array}$$
  (5c) $$\begin{array}{c} R_{\infty }=X_{C}-R_{C}\cos\theta \end{array}$$
12. Once the impedance semicircle is defined, each of the impedances of the triplet define a time constant according to the model described in (1). The proposed time constant $\tau_{actual}$ will be the arithmetic mean of the three time constants. The time constant value can be solved from the real part of the expression (1).
  (6a) C1=Re(ZC,iPx)−Rinf
  (6b) $$\begin{array}{c} C_{2}=R_{0}-R_{\inf} \end{array}$$
  (6c) $$\begin{array}{c} C_{3}=\left(2C_{1}-C_{2}\right)\cos\frac{\alpha \pi }{2} \end{array}$$
  (6d) $$\begin{array}{c} C_{4}=C_{1}-C_{2} \end{array}$$
  (6e) $$\begin{array}{c} C_{5}=\frac{-C_{3}+\sqrt{C_{3}^{2}-4C_{1}C_{4}}}{2C_{1}} \end{array}$$
  (6f) $$\begin{array}{c} \tau =\frac{\sqrt[{1-\alpha }]{C_{5}}}{\omega } \end{array}$$
13. Calculation of the MSE between the impedance values and those obtained from the model. If this error is the smaller until then, the parameters evaluated are proposed as the local solution of the model for the dispersion evaluated. Finally, the procedure loop is closed with the execution of step 9.

### 2.7. Description of the Hardware Validation Study

**Figure 7:**
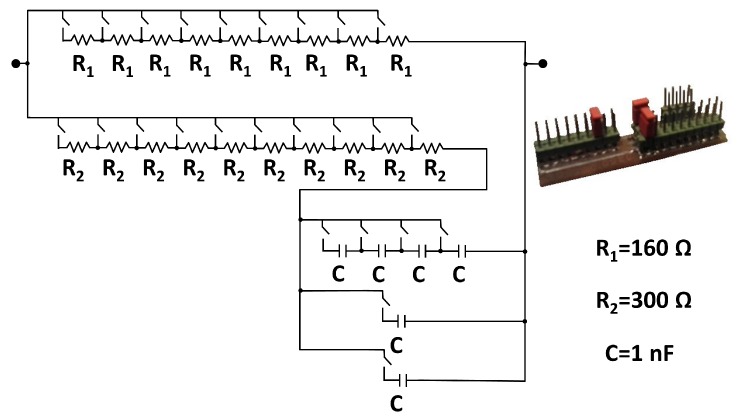
Circuit pattern used in the validation of the sensing subsystem.

Here we refer to the correspondence of bioimpedance data measured with the smart sensor device for a reference pattern. For a first validation of the instrumentation stage of the device, a circuit pattern has been designed that allows emulating different bioimpedance values in a range similar to those that would be obtained in real bioimpedance measurements on subjects with different body characteristics. Figure 7 shows the schematic of the circuit pattern. The smart bioimpedance device will be considered to work correctly according to the design specifications if the error defined as the distance in the complex plane is below 1 % in 22 logarithmically distributed frequencies between 5 kHz and 1 MHz.

For a second validation of the device hardware, a quasi-experimental study has been carried out in a controlled clinical setting on patients with Chronic Obstructive Pulmonary Disease (COPD). This use case has been selected because the loss of body weight and the decrease in muscle mass are some of the most investigated extra-pulmonary characteristics in patients with chronic respiratory diseases [67]. In addition, malnutrition has been recognized as a risk factor associated with increased morbidity, mortality and a deterioration in the quality of life in these patients [68].

The operation of the device was contrasted with a commercial clinical system based on the bioimpedance spectroscopy technique, Body Composition Monitor (BCM) device of Fresenius Medical Care, commonly used for the analysis of body composition. Both devices were used quasi-simultaneously according to the standard measurement procedure with four electrodes in the supine position [1].

The study was approved by the Ethical Committee of the Virgen del Rocío University Hospital of Seville and the participants signed the informed consent. The reproducibility of the measurements was analyzed by comparing the values of the bioimpedance modulus at the aforementioned frequencies. Phase information has not been used in the validation due to the impossibility of having a common phase calibration for the two devices as a consequence of their own electrodes, cables, etc.

To perform the comparative analysis of the prototype measurements with respect to the reference system, statistical parameters such as the mean value and the standard deviation of the error were used. Pearson’s correlation coefficient was also used, considering statistically significant results when *p* < 0.05. Concordance between the two devices was also evaluated using a Bland-Altman diagram. The Matlab programming package (version R2018a) was used to analyze the data.

### 2.8. Description of the Software Validation Study

Here we refer to the model parameter identification obtained through the EC3D algorithm, to be performed by the personal monitoring device, and comparison with other approaches based on the same bioimpedance data.

The proposed model and algorithms have been validated through a set of bioimpedance measurements in a group of peritoneal dialysis (PD) patients. This second use case has been selected because in uremic patients treated with both hemodialysis (HD) and PD, it is very important to assess the amount of fluid excess to determine how much should be removed through ultrafiltration to achieve a desired state of normohydration [69,70]. If fluid excess is not removed long-term consequences can be severe [1,2]. In this sense, bioimpedance analysis has also been successfully applied in nephrology for the identification of the patient’s dry weight and the improvement of cardiovascular management [1,9,69,70,71,72].

The study lasted 24 months and measurements were repeated with the same group of patients during their clinical routine practice. BC estimations were performed by a single PD physician or nurse according to the standard protocol of bioimpedance measurement previously described and using the BCM device of Fresenius Medical Care.

It is considered that an algorithm is more accurate the closer the modeled bioimpedance values are to the measured ones. Thus, in order to quantify the accuracy of the proposed algorithm and its robustness against possible parasitic effects like noise and interferences, a set of parameters related to the error in the modeled bioimpedance values were calculated. This study was performed in the Matlab environment in a standard laptop with 8 GB RAM, a quad-core processor and 2.59 GHz clock rate. For each frequency, the distance between the measured value and the modeled one in the complex bioimpedance plane was considered as a measure of the error, which was normalized with respect to the modulus of measured bioimpedance and multiplied by 100 (relative error in percentage). For each measurement, the following parameters were calculated taking into account the 50 complex bioimpedance values:

1. Mean relative error (MeanRE).
2. Maximum relative error (MaxRE).
3. Standard deviation of the relative error (SDRE).

To evaluate the performance of the algorithm compared to other existing approaches, the previous statistical parameters were evaluated for seven different algorithms described in the literature. For comparison purposes, the algorithm implemented inside the reference device is also included:

1. LSM: Nonlinear Least Squares of the bioimpedance modulus [25].
2. LSC: Least Squares fitting of the admittance with correction of the parasitic capacitance [32].
3. LS: Curve fitting based on Least Squares method [27].
4. LAD: Curve fitting based on Least Absolute Deviation method [27].
5. PSO: Particle-swarm optimization algorithm [29,30].
6. BFO: Bacterial foraging optimization algorithm [30].
7. GA: Genetic algorithm [30,31].
8. BCMa: Algorithm implemented in the Body Composition Monitor (BCM) of Fresenius Medical Care.

The execution time of each of the algorithms was also evaluated in the Matlab environment. For BCMa algorithm, the time elapsed between the completion of the measurement and the identification of the model made by the device was considered. Besides, taking into account that some algorithms may give rise to different parameters every time they run, the identification process and the consequent calculation of the error parameters and execution time were repeated ten times in order to yield a higher statistical significance.

Finally, to obtain an overall statistical representation of the error, the mean, maximum value and standard deviation of the error parameters and execution time were calculated for each algorithm taking into account all the measures and all the identification processes.

## 3. Results

### 3.1. Results of the Hardware Validation Study

Regarding the first evaluation study and following the procedure described in Section 2.7, all configurations of the circuit pattern were evaluated. Previously the device was calibrated following the procedure described in Section 2.4. The error in all the estimations was below 1%, which allowed validating the accuracy and reliability of the smart bioimpedance device. These results are comparable to those obtained in similar systems [73]. This precision has been verified in multiple configurations of the circuit pattern. The use of such a pattern, with resistors and capacitors of known parameters, is necessary to evaluate the estimations, since the actual bioimpedance values of the human body cannot be known a priori.

In the second evaluation study of the device, the recruited volunteers were 12 patients with COPD included in the Respiratory Rehabilitation program of the Virgen del Rocío University Hospital. Table 1 shows the anthropometric characteristics of the volunteers.

**Figure 8:**
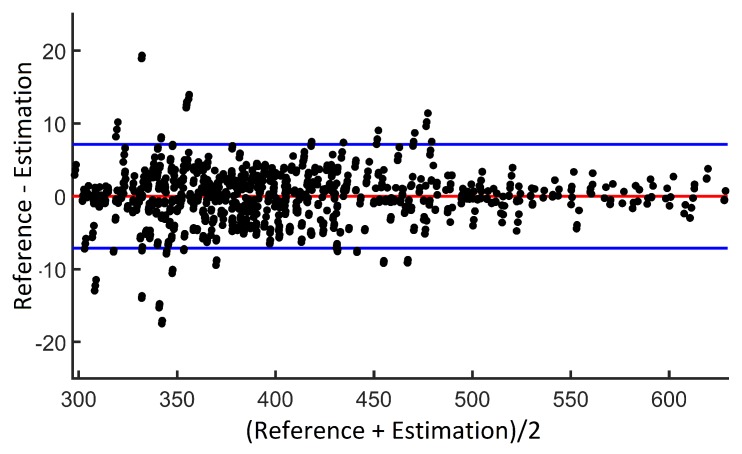
Concordance analysis by Bland-Altman diagram.

The mean error in the bioimpedance modulus was 1.3 Ω with a standard deviation of 2.1 Ω, finding a high correlation between the measurements (r = 0.9984, *p* < 0.0000001). Figure 8 shows the Bland-Altman diagram of the estimations with the two devices: the proposed device and the reference device (BCM from Fresenius Medical Care).

These results show a high agreement with the reference device, with 95% of the measurements in the range of ±7 Ω, which is reasonable since the reference device also has its own precision, and the measurements were performed on the human body where multiple parasitic effects can take place (movements, capacitive effects, differences in the cables that connect the devices with the electrodes, etc.). Comparisons in other studies have shown similar discrepancies when measuring in the human body [74].

### 3.2. Results of the Software Validation Study

Table 2 shows the characteristics of the subjects who participated in the software validation study (49 men/48 women). During the study period, no patient suffered any alteration that involved an exclusion from the group. In total, 193 measurements were performed. For each measurement, the reference BCM device provided 50 complex bioimpedance values between 5 kHz and 1 MHz, parameters of the extended Cole model of one dispersion and an estimation of the BC according to the parameters of the model.

Table 3 shows a comparison of the results obtained in the software validation study described in Section 2.8. EC3D was the most accurate of all the algorithms analyzed, including the reference device algorithm. Regarding MeanRE, which is an indicator that averages the error in the model identification for a multi-frequency measurement, the EC3D algorithm showed the lowest mean value (0.11%), but also the lowest maximum error (0.26%). In this case, the second algorithm that showed the best results was the LAD algorithm. However, since this parameter is averaged, the effects of high and low frequency artefacts may be masked in this average if the identification of bioimpedance values is refined at the intermediate frequencies. Taking this into account, a better indicator of the fit of the artefacts to the model is the MaxRE parameter. Also, with respect to this parameter the EC3D algorithm showed the best performance, with a relative maximum error of 0.68% in the approximation to the bioimpedance values for all the cases analyzed. Regarding this parameter, LAD was also the second best option, but with a relative maximum error of 2.73%.

Regarding the execution time, the recursive algorithms (LSM, LSC, LS and LAD) showed the best performance. The high time obtained for the BFO algorithm is due to the fact that it was very prone to be directed toward local minima of a cost function, with high modeling errors. It was then considered necessary to recursively repeat the identification process until finding the global minimum and acceptable errors.

The cost of the EC3D algorithm to find the suitable solution in all simulations represented an increased runtime with respect to the recursive algorithms. However, the simplicity of the iterative loops allows the algorithm to be employed in a general purpose microprocessor. An implementation of the algorithm (fixed point, variables of two and four bytes, scaled to maintain the accuracy, Taylor series and CORDIC algorithms) required 7687 bytes in the program memory of PIC18LF2431 microprocessor and its execution was carried out in less than 20 s with a 4 MHz clock cycle. Although currently the EC3D algorithm is implemented in the personal monitoring device, these results show the capability of the smart bioimpedance device to identify the parameters of the three-dispersion model. BCM takes a variable time between 20 to 120 s, sometimes even more. The portability of the algorithm in bioimpedance devices demands runtimes not excessively large. The results should be available almost immediately to facilitate the use of the devices during the daily clinical practice. The advantages of the optimization of biomedical algorithms, like the one presented in the paper, are obvious considering the current advances in m-Health and portable medical devices. This emerging paradigm is characterized by very strict operating conditions, which refer to runtimes that allow real-time responses in order to provide an enhanced healthcare supervision of the user.

The results demonstrated the validity of the proposed algorithm, as a method for the precise parameterization of the bioimpedance model. This is a prior process, but essential for the BC estimation since the composition parameters depend directly on the model parameters and an error in its estimation can lead to important errors in nutritional and hydration assessments. It is considered that the use of a model and an algorithm like the one described in the present study, with accurate identification of all bioimpedance values, can provide a BC estimation more robust against possible artefacts and disturbances. This assumption can be corroborated by considering the estimates made by the reference device in the cases where a remarkable imbalance appeared between bioimpedance measurements and values derived from the BCM Cole model.

**Figure 9:**
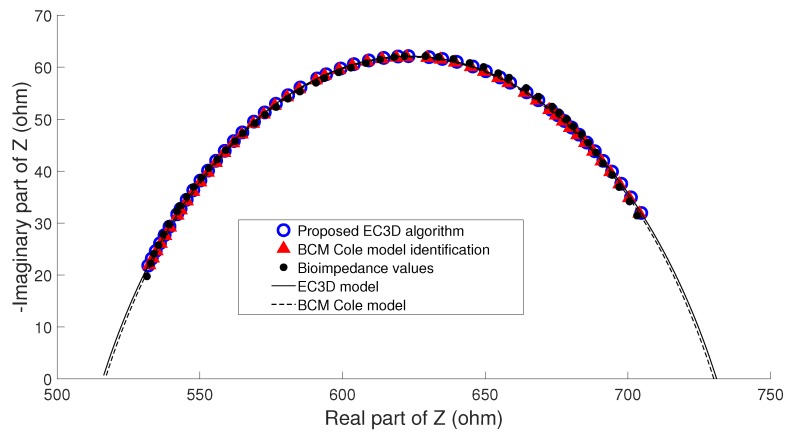
Cole diagram of patient in example A (normal situation).

**Figure 10:**
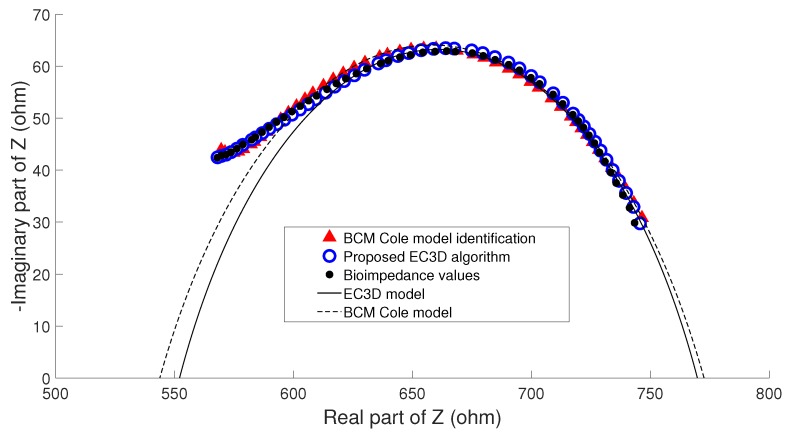
Cole diagram of patient in example A (high-frequency artefact).

**Figure 11:**
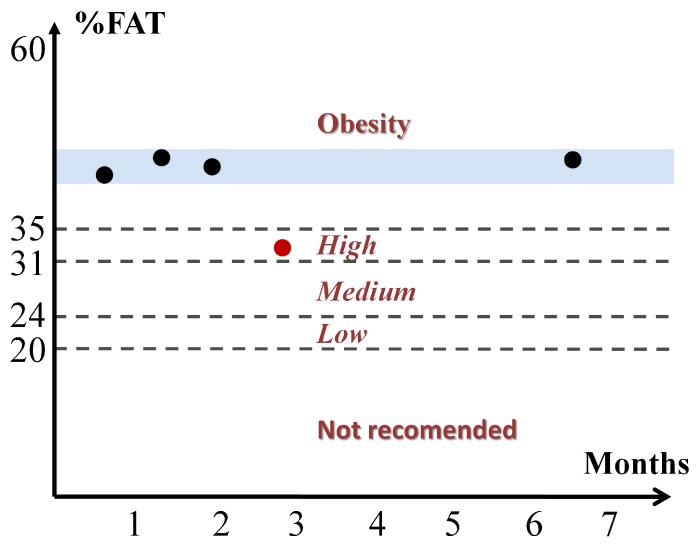
Classification by percentage of FAT [75] of patient in example B.

**Figure 12:**
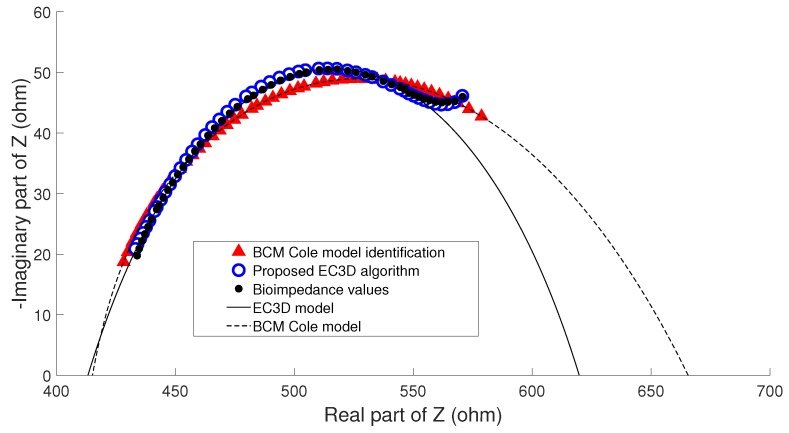
Cole diagram of patient in example C (low-frequency artefact).

**Figure 13:**
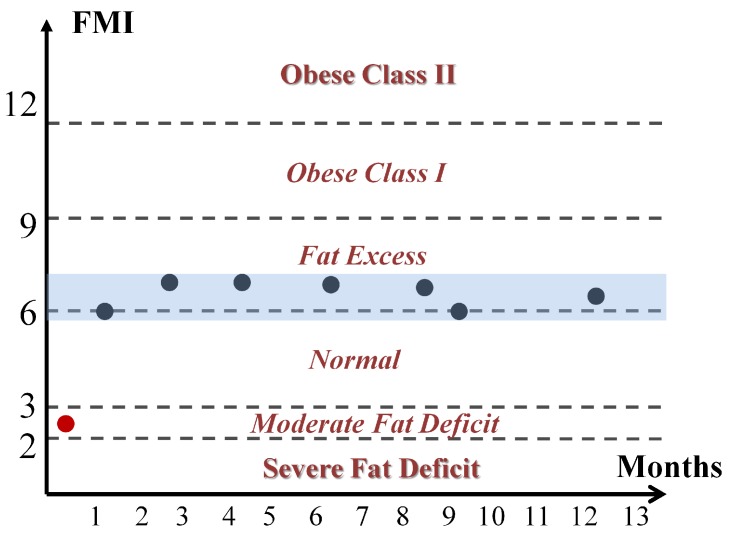
Classification by Fat Mass Index [76] of patient in example C.

Due to the fact that BCM estimates were not made with another method that could serve as a reference, it was not possible to consider a priori that an error occurred when performing the parameter identification. However, taking into account that in the clinical study it is possible to develop an analysis of the evolution of patient status, other measurements may be used as the reference for the Cole parameters. To address this issue the hypothesis considered is based on the fact that if the clinical evaluation of patients (weight, nutritional assessment, detection of edema) showed no change between consecutive measurements, any change in the physical condition of a patient can only develop gradually, so that the nutritional status of the patient cannot undergo significant change between two consecutive estimates of BC performed in a short period of time. In this case, the term nutritional status of the patient stands for his/her amount of fat and lean, and not the state of hydration, since it can undergo significant changes in patients under renal replacement therapy in both hemodialysis and peritoneal dialysis. This clinical analysis was performed with the help of nephrology specialists. Three examples that highlight the main ideas of the hypothesis are described as follows:

- **Example A**: In the majority of the cases studied, no parasitic effect was observed in the bioimpedance measurements at high or low frequency, which could be verified by analyzing the results of the proposed EC3D algorithm. As an example, Figure 9 shows the conventional cole diagram of a bioimpedance measurement performed on a voluntary. In this case, both the BCM extended Cole model and the proposed EC3D algorithm agreed in the process of identifying the bioimpedance model. $R_{0}$ and $R_{\inf}$ were very close ($R_{0,BCM}=730.1\;\Omega$, $R_{0,EC3D}=731.1\;\Omega$, $R_{\inf,BCM}=516.8\;\Omega$, $R_{\inf,EC3D}=515.9\;\Omega$), and therefore, the estimation of body composition.
- **Example B**: Figure 10 shows bioimpedance values of a patient measurement on a Cole diagram. Bioimpedance values show a hook up effect at high frequency. As mentioned in Section 2.4, a possible cause of this behavior may be the parasitic capacitance established between the human body and the ground plane [63]. This effect was corrected by the BCM extended Cole model incorporating a major phase delay ($T_{d}=5.27$ nseg). However, it can be observed that bioimpedance values do not fit the model at both high and intermediate frequencies. On the other hand, the proposed EC3D algorithm matched this disturbance effect at high frequency with an additional dispersion, obtaining a better fit to the bioimpedance values. The result was a discrepancy in the identification of the parameters of the bioimpedance model ($R_{0,BCM}=772.6\;\Omega$, $R_{0,EC3D}=769.8\;\Omega$, $R_{\inf,BCM}=543.8\;\Omega$, $R_{\inf,EC3D}=552.1\;\Omega$), which also resulted in differences in the estimation of body composition.
  The evolution of the nutritional status of the patient in example B according to the percentage of fat is shown in Figure 11, which was assessed by means of bioimpedance measurements. The dots in the figure indicate the value of the percentage of fat in the time instants in which the measurements were made. Dashed lines allow to visually classify the state of the patient according to [75]. The dot with different color (measurement 4) shows a significant variation in the nutritional status with respect to the other estimations, which are grouped on the colored band.
- **Example C**: Figure 12 also shows bioimpedance values of another patient measurement. In this other case, a hook up effect appears at low frequency. Although this type of bioimpedance behavior moves away from the expected response of a body bioimpedance measurement, as was mentioned in Section 2.4, this type of phenomenon can occur when there is a bad contact between the electrode and the skin, which results in a high value of the electrode impedance [58,59,61,62]. Our experiences corroborate this fact, since when during the experiments on patients hook up effects were observed at low frequency, it was found that in many cases one of the electrodes was not well attached to the skin. The replacement of the electrode and the repetition of the measurement solved the problem. Even so, some of the cases were not detected at the time of the measurement (one of them is Example C), which indicates the need to incorporate a method of detection of such artefacts in the measurement equipment and/or a procedure to correct them. In this case, the BCM model tried to approximate the bioimpedance values by a large phase lead ($T_{d}=-6.12$ nseg), but a relevant mismatch is observed in all the frequency range. The proposed EC3D algorithm corrected this disturbance effect with an additional dispersion at low frequency, also getting a good fit to the bioimpedance values. In this case, a large difference was found in the value of $R_{0}$ identified by each model ($R_{0,BCM}=665.6\;\Omega$, $R_{0,EC3D}=619.8\;\Omega$, $R_{\inf,BCM}=415.1\;\Omega$, $R_{\inf,EC3D}=413.2\;\Omega$), giving rise to differences also in body composition.
  The same considerations of Figure 11 can also be applied to the case of Figure 13, which shows the evolution of the patient in example C according to the Fat Mass Index described in [76]. In this case, measurement 1 showed a significant variation with respect to the rest of the measurements.

Without any further reference method with which to compare and considering the above hypothesis, the validity of the proposed algorithm was evaluated using the estimation of ECW, ICW, and TBW of measurements performed close in time. Table 4 shows as an example of these considerations a comparison of ECW, ICW and TBW provided by the BCM device for patient B and those derived from the application of the parameters obtained with the proposed method ($ECW_{m}$, $ICW_{m}$ and $TBW_{m}$) to the model described in [3]. In all the bioimpedance measurements of this work, the BC model described in [3] approximated with a high degree of accuracy the values predicted by the BCM, with an error lower than 1% when the Cole parameters provided by the device were used, validating its use as BC model for the comparison.

Measurement 1 in Table 4 corresponds to the abnormality detected in the model, and in this case, the application of the proposed algorithm provides very similar values for the BC parameters obtained in the other measurements. These parameters, and especially ICW, are directly related to the nutritional status of the patient, and, taking into account the clinical information of the patients, according to which the patients maintained their physiological and nutritional status throughout the study, it is expected that the value of the ECW, ICW and TWB parameters in the first measurements were similar to the rest. Assuming this consideration, the proposed EC3D algorithm showed the closest results to the expected values of the parameters in this and other similar cases, showing the validity of the method.

## 4. Conclusions

A new smart bioimpedance spectroscopy device has been investigated, developed and validated for its application to the estimation of body composition.

The main novelties presented by the device compared to commercial systems are its low cost, thanks to a hardware based on standardized electronic components and the use of a single multiplier to measure both in-phase and quadrature components of bioimpedance. Another advantage of the proposed device is its capability to be controlled remotely through bidirectional wireless communications. The distributed design of the system and the ability to personalize the device both in operation and in frequency selection allows the system to be used in other bioimpedance applications different from the estimation of body composition. It is worth mentioning the semi-automatic procedure proposed for the calibration of the device which allows the revision and adjustment of the hardware to maintain a clinical level of accuracy over time.

The present work has also contributed a new algorithm for the identification of the Cole model parameters which is able to correct and avoid many of the disturbances that can occur in a bioimpedance measurement. When bioimpedance measurements are affected by some kind of perturbation, noise or parasitic effect, the obtained parameters can be affected, thereby inducing significant errors in the body composition estimation. The proposed algorithm solves a Cole model with three dispersions, not so commonly employed in the literature, to which it is added the effect of a delay (or lead) in phase linear with frequency with respect to the typical phase delay of the device (usually corrected by factory calibration).

The results obtained in a hardware validation study have shown the accuracy and feasibility of the proposed solution. The software validation results have shown the robustness of the algorithm against these parasitic effects. A comparison with other existing algorithms highlights the precision of the proposed method against other proposals with a relative maximum error of 0.68%. Moreover, another advantage of the method is its simplicity, which allows the algorithm to be implemented in general purpose microprocessors using a quasi-analytic solution that takes advantage of the particular bioimpedance frequency behavior. Another comparison with respect the results obtained by a bioimpedance spectroscopy device validated for clinical use showed a good correspondence with the reference device, except in those cases where measurements were altered by some kind of parasitic effect. In these exceptional cases, the proposed algorithm provided better approximations to the bioimpedance values and an analysis of the clinical evolution of the patients confirmed the appropriateness of using the parameters of the proposed model.

The results encourage further research in this direction, highlighting the importance of medical assessment (technical or specialist) when interpreting any bioimpedance-related data, and the need to incorporate methods to analyze the measurements in biomedical devices, to warn about abnormalities or errors detected on-site, either by analyzing the bioimpedance values or by analyzing the evolution of the estimates of the patients. The origin of these alterations can be multiple (defects in the electrodes or inappropriate placement on the body, the patient is not in the proper position, not enough time is spent so that the volume of fluid in the different compartments could not be stabilized, etc.) and its causes must also be analyzed. In these cases, it may be advisable to make a second estimate repeating the whole measurement process (cleaning the skin, electrode placement, etc.) in order to compare the results obtained. The proposed algorithm provides a way to address this issue, detecting these anomalies when they are produced, which can be used to trigger a warning message to repeat the measurement, and, in cases in which these effects remain, providing an improved approximation to the Cole model parameters.

## Figures and Tables

**Table 1:** Patients Anthropometric Characteristics of the Hardware Validation Study.

	Mens	Women
Number of volunteers	9	3
	**Average Value**	**Standard Deviation**
Weight (kg)	95.8	18.6
Age (years)	60.6	7.6
Height (cm)	163.5	6.1

**Table 2:** Anthropometric Characteristics of the Software Validation Study Patients.

	Min	Medium	Max	σ
Age (years)	31	61.8	86	15.6
Weight (kg)	39	71.9	123.5	16.7
Height (cm)	140	161.7	191	9.6
Body Mass Index	17.6	27.4	42.4	5.1

**Table 3:** Error Analysis of EC3D and Comparison with other Existing Algoritms.

	*MeanRE*			*MaxRE*			*SDRE*			Execution Time (sec)		
	Mean	Max	SD	Mean	Max	SD	Mean	Max	SD	Mean	Max	SD
EC3D	0.11	0.26	0.05	0.29	0.68	0.14	0.06	0.15	0.03	1.92	1.92	0.00
LSM	0.38	1.51	0.23	1.73	8.70	1.26	0.39	2.02	0.31	0.04	0.34	0.02
LSC	0.54	1.92	0.13	3.13	12.25	0.80	0.63	2.33	0.16	0.03	0.19	0.01
LS	0.37	1.01	0.15	0.87	3.60	0.58	0.21	0.72	0.11	0.01	0.15	0.01
LAD	0.15	0.46	0.07	0.51	2.73	0.38	0.10	0.55	0.07	0.02	0.15	0.01
PSO	0.19	1.51	0.22	0.55	4.00	0.59	0.09	0.61	0.10	0.55	0.89	0.02
BFO	0.73	5.99	0.74	1.98	18.01	2.26	0.40	3.98	0.51	71.36	929.05	83.30
GA	0.28	0.75	0.11	0.80	4.40	0.35	0.15	0.88	0.07	5.83	6.82	0.15
BCMa	0.14	0.51	0.08	0.44	1.50	0.24	0.08	0.25	0.04	70.64	148.5	29.98

**Table 4:** Comparison of BC parameters in the case of patient B throughout the study.

(Liters)	*ECW*	*ECW_m_*	*ICW*	*ICW_m_*	*TBW*	*TBW_m_*
Meas. 1	15.5	16.7	27.3	19.9	42.7	36.6
Meas. 2	18.8	18.8	20.5	20.4	39.3	39.3
Meas. 3	17	17.1	18.6	18.6	35.7	35.7
Meas. 4	17.3	17.3	19.9	19.9	37.2	37.2
Meas. 5	15.9	15.9	19.4	19.3	35.3	35.3
Meas. 6	16.3	16.3	19.8	19.8	36.2	36.1
Meas. 7	16.4	16.4	20.3	20.3	36.8	36.7

## Abbreviations

The following abbreviations are used in this manuscript:

BC	Body Composition
BCM	Body Composition Monitor
BCMa	BCM device algorithm
BFO	Bacterial foraging optimization algorithm
CMRR	Common-Mode Rejection Ratio
COPD	Chronic Obstructive Pulmonary Disease
DDS	Direct Digital Frequency Synthesis
EC3D	Extended Cole model with 3 Dispersions algorithm
ECW	Extracellular Water
FFM	Fat Free Mass
FM	Fat Mass
GA	Genetic algorithm
HD	Hemodialysis
ICW	Intracellular Water
LAD	Curve fitting based on Least Absolute Deviation method
LS	Curve fitting based on Least Squares method
LSC	Least Squares fitting of the admittance with correction of the parasitic capacitance
LSM	Nonlinear Least Squares of the bioimpedance modulus
MaxRE	Maximum relative error
MeanRE	Mean relative error
MSE	Mean square error
NLLS	Nonlinear Least Squares
OpAmps	Operational amplifiers
PD	Peritoneal Dialysis
PSO	Particle-swarm optimization algorithm
SDRE	Standard deviation of the relative error
TBW	Total Body Water
